# Long-Term Fluoxetine Administration Causes Substantial Lipidome Alteration of the Juvenile Macaque Brain

**DOI:** 10.3390/ijms22158089

**Published:** 2021-07-28

**Authors:** Anna Tkachev, Elena Stekolshchikova, Daniil M. Bobrovskiy, Nickolay Anikanov, Polina Ogurtsova, Dong Ik Park, Anja K. E. Horn, Daria Petrova, Ekaterina Khrameeva, Mari S. Golub, Christoph W. Turck, Philipp Khaitovich

**Affiliations:** 1 V. Zelman Center for Neurobiology and Brain Restoration, Skolkovo Institute of Science and Technology, 121205 Moscow, Russia; anna.tkachev@skolkovotech.ru (A.T.); E.Stekolschikova@skoltech.ru (E.S.); koenzyme@mail.ru (N.A.); P.Ogurtsova@skoltech.ru (P.O.); D.Petrova@skoltech.ru (D.P.); 2 Faculty of Bioengineering and Bioinformatics, Moscow State University, 119234 Moscow, Russia; daniil.bobrovsky@fbb.msu.ru; 3 Proteomics and Biomarkers, Max Planck Institute of Psychiatry, 80804 Munich, Germany; park@biomed.au.dk; 4 Institute of Anatomy and Cell Biology, Ludwig-Maximilians University, 80336 Munich, Germany; Anja.Bochtler@med.uni-muenchen.de; 5 Center of Life Sciences, Skolkovo Institute of Science and Technology, 121205 Moscow, Russia; 6 California National Primate Research Center, University of California, Davis, CA 95616, USA

**Keywords:** fluoxetine, antidepressant, lipidomics, lipids, PUFA, transcriptomics, depression, macaca mulatta

## Abstract

Fluoxetine is an antidepressant commonly prescribed not only to adults but also to children for the treatment of depression, obsessive-compulsive disorder, and neurodevelopmental disorders. The adverse effects of the long-term treatment reported in some patients, especially in younger individuals, call for a detailed investigation of molecular alterations induced by fluoxetine treatment. Two-year fluoxetine administration to juvenile macaques revealed effects on impulsivity, sleep, social interaction, and peripheral metabolites. Here, we built upon this work by assessing residual effects of fluoxetine administration on the expression of genes and abundance of lipids and polar metabolites in the prelimbic cortex of 10 treated and 11 control macaques representing two monoamine oxidase A (*MAOA*) genotypes. Analysis of 8871 mRNA transcripts, 3608 lipids, and 1829 polar metabolites revealed substantial alterations of the brain lipid content, including significant abundance changes of 106 lipid features, accompanied by subtle changes in gene expression. Lipid alterations in the drug-treated animals were most evident for polyunsaturated fatty acids (PUFAs). A decrease in PUFAs levels was observed in all quantified lipid classes excluding sphingolipids, which do not usually contain PUFAs, suggesting systemic changes in fatty acid metabolism. Furthermore, the residual effect of the drug on lipid abundances was more pronounced in macaques carrying the MAOA-L genotype, mirroring reported behavioral effects of the treatment. We speculate that a decrease in PUFAs may be associated with adverse effects in depressive patients and could potentially account for the variation in individual response to fluoxetine in young people.

## 1. Introduction

Psychiatric disorders in young people, including depression, attention deficit hyperactivity disorder, autism, mental retardation, and obsessive-compulsive disorder, are commonly treated with antidepressant medications. One such drug is fluoxetine, a selective serotonin reuptake inhibitor (SSRI) that is used to manage behavioral symptoms in children and young adolescents. Although treatment efficacy of fluoxetine in children has been demonstrated [1,2,3,4,5,6,7,8,9,10,11,12], individual variability in response, including potential long-term adverse effects observed in adolescents [13,14,15], has highlighted the need to examine the effects of this drug on the developing brain.

As is the case for any psychoactive drug, possible adverse effects on brain development with long-term consequences for health and behavior need to be taken into account when administered to young people [16,17]. Due to ethical reasons, long-term studies with psychoactive drugs in young people cannot be carried out, but these drugs can be investigated in animals with the goal to delineate possible adverse effects. One study has demonstrated an increase in depressive- and anxiety-like behaviors in adulthood when rodents were subjected to fluoxetine treatment in early life [18], implicating brain developmental effects of the drug. Research carried out in non-human primates, however, is far more relevant for studying the consequences of chronic psychoactive drug administration on neuroanatomy and behavior, since these animals have a prolonged cognitive development that is similar to humans [19,20,21,22,23]. In addition, and of relevance to our studies, nonhuman primates exhibit polymorphisms in genes that are related to psychiatric phenotypes and the psychoactive drug response.

Several reports by us and others [17,24,25,26,27] on the effects of fluoxetine in juvenile macaques have already provided valuable insights into affected brain structures and behavior. During two years of daily dosing, the results of our studies demonstrated effects on impulsivity [26], bone growth [28], sleep [24], and social interaction [25,29], and have delineated peripheral metabolite biomarkers of drug action [26]. Some of the behavioral effects persisted after discontinuation of dosing. Furthermore, effects of fluoxetine on gene expression and lipid metabolism were reported in rodents [30,31,32], accompanied by reports of metabolic alterations in human adults receiving fluoxetine treatment [33]. Together, these studies suggest systemic alterations in brain and body metabolism and gene regulation induced by long-term fluoxetine treatment. To address this, in the current study we have extended our investigations on long-term chronic fluoxetine administration effects in juvenile macaques to multi-omics analyses of brain tissue collected one year after termination of treatment. Specifically, we were interested in gene expression and lipid and polar metabolite level alterations in the prelimbic cortex (PLC), a subregion of the medial prefrontal cortex that has important roles in working memory and contextual processing. Our focus on this brain area was prompted by increased binding potential of SERT gene one year after discontinuation of a two-year-long fluoxetine treatment in juvenile macaques [34] and suggested effects of the drug on dendritic spine synapse density [27].

## 2. Results

**Figure 1:**
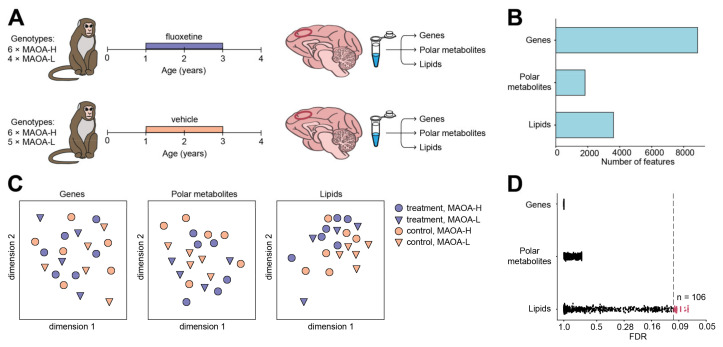
(**A**) Schema of experimental design. (**B**) Number of quantified genes, polar metabolites, and lipids. (**C**) Multi-dimensional scaling plots visualizing variation among samples calculated based on abundance levels of 8871 genes, 1829 polar metabolites, and 3608 lipids. Colors correspond to treatment status, shapes to the *MAOA* genotype. (**D**) Distribution of t-test FDR values calculated for 8871 genes, 1829 polar metabolites, and 3608 lipids in the comparison between treated and control animals. Dashed line corresponds to FDR cutoff of 10%. The number of lipids passing this FDR cutoff (red) is marked on the right. The identified number of lipids passing the cutoff is greater than expected by chance (permutations, *p* = 0.008).

We assessed alterations of gene expression, polar metabolite, and lipid abundance in the PLC of macaques treated with fluoxetine using RNA-sequencing (RNA-seq), Fourier-transform ion cyclotron resonance mass spectrometry (FT-ICR-MS), and high precision mass spectrometry coupled with liquid chromatography (LC-MS), respectively (Figure 1A). The fluoxetine and vehicle administration began at one year of age, which is equivalent to 4–6 years of age in humans, and continued uninterrupted for two years, followed by a one year post-dosing period that ended at four years of age, just before puberty [35]. Our analysis included 10 monkeys treated with fluoxetine and 11 control monkeys administered with a vehicle (Table S1). Both treated and control groups were made up of male animals randomized for factors such as age, size, cage location, and were represented by two distinct genotypes with high (MAOA-H, *n* = 12) and low (MAOA-L, *n* = 9) transcription rates of the monoamine oxidase A (*MAOA*) gene (Figure 1A; Table S1).

RNA-seq yielded quantitative gene expression measurements for 8871 protein-coding genes annotated in the macaque genome (Table S2). Mass spectrometry analyses generated abundances for 1829 polar metabolite and 3608 lipid features (Tables S3 and S4), with 514 polar metabolite features and 373 lipid features putatively annotated (Figure 1B; Tables S5 and S6). Of these 373 lipid features, 78 (21%) further generated informative MS/MS fragmentation patterns, all 78 of them matching the putative annotations (Table S6). Visualization of variation among samples based on these three data types did not reveal outliers and indicated the separation of treated and control macaques at the lipid abundance level, but not at the gene and polar metabolite levels (Figure 1C).

**Figure 2:**
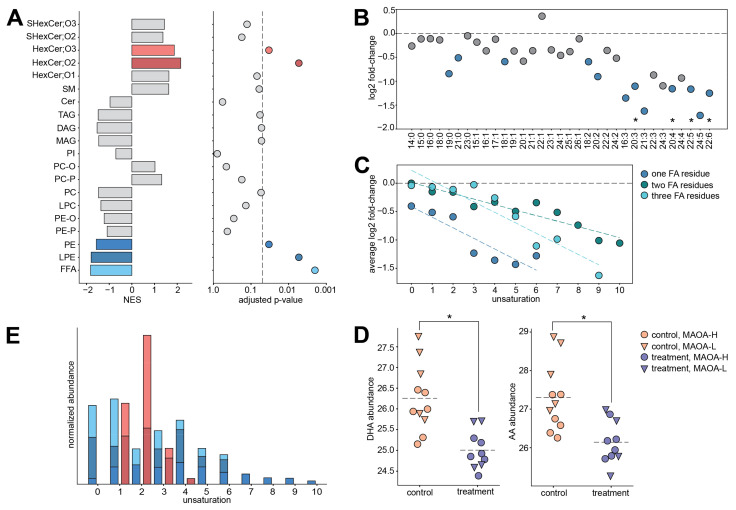
(**A**) Lipid class enrichment analysis results based on the comparison between treated and control animals. Left: normalized enrichment scores (NES). Positive NES corresponds to an increase in the lipid abundances in treated animals, and negative NES corresponds to decrease. Colors mark lipid classes demonstrating significant enrichment in the treatment-control differences. Right: adjusted enrichment *p*-values. The dashed line corresponds to the adjusted *p*-value cutoff of 0.05. (**B**) Base two log-transformed fold change (log2 FC) values calculated between treated and control animals for lipids in free fatty acid (FFA) class. *X*-axis labels indicate fatty acid chain length and number of double bonds. FFAs are ordered by increasing unsaturation. Blue symbols mark compounds with nominal *t*-test *p* < 0.05. Asterisks mark statistically significant compounds (*t*-test; FDR = 10%, permutations *p* = 0.0008). The dashed line indicates log2 FC = 0. (**C**) Log2 FC values calculated between treated and control animals, averaged for all the lipids with the same level of unsaturation (number of double bonds). Lipids were first separated into groups based on the number of fatty acid residues, one (dark blue), two (green), or three (light blue), and sphingolipids were excluded. The black dashed line indicates log2 FC = 0. Colored dashed lines indicate linear regression lines fitted to each group (*p* = 0.0054, 0.54 × 10^−5^ and 0.00022 for lipid classes containing one, two and three fatty acid residues, respectively). (**D**) Abundance levels of docosahexaenoic acid (DHA) and arachidonic acid (AA) in treated and control animals. Symbols represent individual samples. Colors correspond to treatment status, shapes to the *MAOA* genotype. Asterisks mark statistically significant differences between treatment and control (*t*-test; FDR = 10%, permutations *p* = 0.0008). (**E**) The cumulative abundances of lipids contained in FFA, LPE, PE, HexCer;O2, and HexCer;O3 lipid classes (colors as in panel A) for each level of unsaturation (number of double bonds per compound). Cumulative abundances were normalized between the lipid classes.

Statistical analysis revealed 106 lipid features (treatment-associated lipids) showing significant abundance differences between fluoxetine-treated and control monkeys (permutations, *p* = 0.008; Benjamini-Hochberg adjusted FDR = 10%). By contrast, the treatment effect was substantially weaker at the gene expression and polar metabolite abundance levels, and statistical effects were considered too low to define any reasonable false-discovery rate (FDR) threshold for treatment-associated genes or metabolites (Figure 1D; Tables S5 and S7; minimal observed *q*-value = 0.9986 and 0.68740 for gene expression and polar metabolite levels, respectively). We further conducted group-based analysis assessing the significance of the treatment effect at the level of lipid classes. This approach revealed significant abundance differences for five lipid classes: free fatty acids (FFA), phosphatidylethanolamines (PE), lysophosphatidylethanolamines (LPE), and hexosylceramides (HexCer;O2 and HexCer;O3) (Figure 2A and Figure S1; Gene Set Enrichment Analysis, adjusted *p* < 0.05, Table S8). Notably, lipids within a class showed coordinated treatment response, exhibited by the abundance of HexCer;O2 and HexCer;O3 lipids increasing in the PLC of the treated monkeys and FFA, LPE, and PE lipids decreasing as a result of the treatment (Figure S2).

The effect of fluoxetine was not uniform within a lipid class, but depended strongly on its fatty acid residue composition. Specifically, lipids containing fatty acids with multiple double bonds, or polyunsaturated fatty acids (PUFAs), were affected by the treatment to a greater extent. This effect was most obvious for free fatty acids (Figure 2B), but also evident for the other detected glycerophospho- and glycero-lipid classes (F-test for linear regression, *p* = 0.0054, 0.00004, and 0.00022 for lipid classes containing one, two, and three fatty acid residues, respectively; Figure 2C, Figure S3). Furthermore, the stronger effect of fluoxetine for unsaturated FFAs could not be explained by underlying distribution of abundance levels or fatty acid chain lengths (F-test for linear regression, *p =* 0.35 and 0.26 for abundance and chain length, respectively; Figure S4). Notably, the two PUFAs constituting up to 25% of all fatty acid content in the brain, docosahexaenoic acid (DHA, FFA 22:6) and arachidonic acid (AA, FFA 20:4), as well as their close counterparts AA-containing monoacylglycerol (MAG 20:4), docosapentaenoic acid (FFA 22:5), and FFA 20:3, differed significantly between treated and control animals at the individual compound level (Benjamini-Hochberg adjusted FDR = 10%; Figure 2D, Table S6). None of the other putatively annotated lipids were found to be significantly altered between treated and control animals at the individual compound level (Benjamini-Hochberg adjusted FDR = 10%; Table S6). In contrast to glycerophospholipids and glycerolipids, which contain substantial amounts of PUFA residues, lipid classes depleted in PUFAs, HexCer;O2 and HexCer;O3, increased significantly as a result of the treatment (Figure 2A,E). In a previous study, we had likewise found lower blood and cerebrospinal fluid unesterified AA levels during treatment in the same fluoxetine-treated animals compared to controls [26]. Furthermore, the reported analysis performed on blood and cerebrospinal fluid samples following one year of daily dosing also revealed a significant treatment-by-*MAOA*-genotype interaction for AA levels [26].

**Figure 3:**
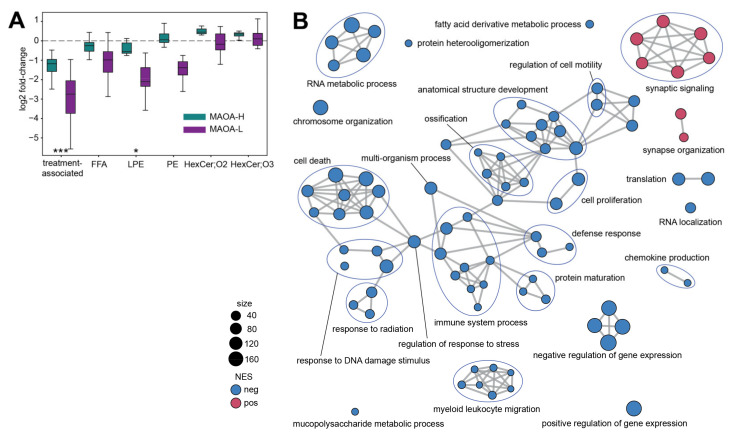
(**A**) Base two log-transformed fold-change (log2 FC) values calculated between treated and control animals carrying MAOA-H (green) and MAOA-L (purple) genotype. X-axis labels indicate lipids used in the comparison: 106 treatment-associated lipids and lipids contained in treatment-affected lipid classes: FFA, LPE, PE, HexCer;O2, and HexCer;O3. Boxes show median log2 FC values and interquartile interval. Error bars extend according to Tukey’s original boxplot definition. Asterisks indicate enrichment in lipids showing treatment-by-MAOA genotype interaction (hypergeometric test, *** *p* < 0.001, * *p* < 0.05). (**B**) Gene ontology (GO) terms enriched in gene expression differences between treated and control animals (GSEA, adjusted *p* < 0.05). One point indicates one GO term. Colors show negative (blue) and positive (red) normalized enrichment score values, which correspond to the dominant direction of expression change between treated and control animals within the GO term. Symbol size is proportional to the number of genes in the GO term. Edges connect functionally related GO terms.

Interaction between fluoxetine treatment and *MAOA* genotype has been shown not only for circulating metabolite levels in juvenile macaques, but for behavioral responses, as well [26,35]. In line with this observation, compared to the average, the 106 treatment-associated lipids showed an increased number of treatment-by-genotype interactions identified using ANOVA (enrichment *p* < 10^−7^, Figure 3A; Table S6). In agreement with results obtained at the lipid class level (Figure 2A), most of the treatment-associated lipids showed reduced abundances in fluoxetine-treated animals. Accordingly, monkeys carrying MAOA-L genotype displayed a larger decrease of FFA, LPE, and PE lipid levels after fluoxetine treatment (Figure 3A).

While we did not detect significant gene expression and polar metabolite abundance differences in the PLC of fluoxetine-treated macaques, below-the-threshold effects might still be informative. First, we tested whether genes that encode 131 detected enzymes catalyzing reactions involving lipids showed more expression differences compared to the rest. We indeed found a small but significant shift towards larger differences between treated and control macaques for the enzyme gene group (permutations, *p* = 0.0239; Table S9). Next, using group-based analysis designed to reveal sub-threshold effects, we identified significant treatment effects at the gene expression level, but none for polar metabolites. Specifically, 87 gene groups defined using Gene Ontology (GO) biological process terms differed in fluoxetine-treated macaques (Gene Set Enrichment Analysis, adjusted *p* < 0.05; Figure 3B and Figure S5; Table S10). Notably, identified gene groups separated based on the direction of expression difference. All increased terms related to synaptic functions, including synaptic organization and signaling (Figure 3B and Figure S5). By contrast, decreased terms were mainly associated with immune response, as well as apoptosis, RNA processing, and tissue proliferation (Figure 3B and Figure S5; Table S10).

## 3. Discussion

Our study provides an intriguing opportunity to assess the effects of long-term fluoxetine administration at three levels of molecular endophenotype: brain transcriptome, metabolome, and lipidome. The results show that, while fluoxetine administration substantially alters the brain lipid composition, the effects at gene expression and polar metabolite abundance levels were subtle and non-detectable, respectively.

Reduction of the PUFAs abundance in treated animals’ brains constituted the main trend of detected lipidome alterations. This effect was most prominent for FFAs—a lipid class representing fatty acid molecules not attached to any lipid “head” group, such as glycerol or sphingosine. Nonetheless, the decrease in PUFAs content was evident for all detected lipid classes containing PUFA residues. While previous studies did not conduct the lipidome analysis of fluoxetine-treated animals, an investigation of fatty acid levels in the rat prefrontal cortex identified treatment-dependent changes in PUFAs abundance for DHA [36] and docosapentaenoic acid [37]. Further, DHA supplementation was shown to influence rhesus macaque brain development enhancing motor and orientation control in early postnatal period [38]. At the level of lipid metabolism, gene expression studies have reported alterations for myelination genes in the rat hippocampus [31], as well as fatty acid elongation genes in zebrafish brain [39].

There is a growing recognition of the role played by PUFAs in the pathogenesis of psychiatric disorders, including mood disorders [40]. Studies have shown a decrease of DHA in post-mortem tissues of the orbitofrontal cortex of patients with bipolar disorder [41], major depressive disorder [42], as well as a decrease of both DHA and AA in patients with schizophrenia [43]. Further, DHA concentrations decreased in the prefrontal cortex of major depressive disorder patients [44]. In blood, patients suffering from depression displayed reduced omega-3 fatty acids levels, including DHA [45,46]. Moreover, omega-3 supplementation was suggested to improve depressive symptoms [47,48], while dietary omega-3 PUFA deprivation induced depressive-like behaviors in rodents [49,50,51] coupled with a decrease in prefrontal cortex DHA levels [49]. While brain PUFAs levels are relatively stable in adulthood, the brain undergoes major changes in fatty acid content in childhood, including accretion of DHA [52,53], highlighting the risk of brain PUFAs metabolism abnormalities during this period. Together with the fluoxetine treatment-associated decrease in PUFAs content of the juvenile macaque brain found in our study, these observations suggest a potential risk of fluoxetine treatment effect on the developing brain.

While our study provides no direct clues towards the possible mechanism causing treatment-induced PUFAs abundance decrease, a reduction in PUFAs biosynthesis in the liver is a possible scenario. The brain contains large amounts of long-chain PUFAs compared to non-nervous tissue, which are not locally synthesized, but predominantly transported to the brain via the blood–brain barrier [54]. The plasma pool of long-chain PUFAs, such as AA and DHA, are either derived from the diet, or synthesized in the liver from their precursors 18:2n-6 linoleic and18:3n-3 alpha-linolenic acid [55,56,57,58,59]. Multiple studies have indicated changes in liver functionality in fluoxetine-treated animals that might result in a decrease of PUFAs synthesis. Specifically, fluoxetine treatment induced lipid metabolism abnormalities and lipid accumulation in mouse liver in the form of triglycerides [33,60], caused liver damage, and promoted apoptotic signaling in rat liver [61,62]. Moreover, fluoxetine was shown to impact sterol regulatory element binding proteins (SREBP) transcription factors in hepatocytes [60], which are essential for the regulation of enzymes used in PUFAs synthesis [63,64,65]. Additionally, in a previous study we detected a reduction of AA in the blood plasma of fluoxetine-treated macaques [26], further supporting the hypothesis that fluoxetine can introduce organism-wide fatty acid metabolism alterations. While we cannot make conclusions about causality, our previous studies including the same macaque individuals [24,29,35] demonstrated that these fluoxetine-related metabolic alterations were accompanied by behavioral alterations as well.

We showed that macaques carrying MAOA-H and MAOA-L genotypes visibly differed in the amplitude of treatment-dependent lipidome alterations, with MAOA-L animals showing greater effects. These results align with our previous report of an emotional response reduction in response to fluoxetine treatment in MAOA-L but not in MAOA-H macaques [29], and residual fluoxetine post-treatment effects on brain serotonin transporter binding potential being especially pronounced in MAOA-L monkeys [34]. Intriguingly, difference in the genotype-dependent fluoxetine effect amplitude identified in rhesus macaques parallels the differences reported for behavioral traits in both humans and macaques. Specifically, human males with the low-activity *MAOA* variant who were exposed to adversity in childhood were more likely to show aggression [66], as well as anti-social behavior in adulthood [67]. Similarly, other studies have linked high aggression and MAOA-L genotype [68,69]. The genotype-dependent difference in fluoxetine effect we report on both lipid and behavioral levels are in agreement with possible individual side-effects of the drug treatment described for younger patients [13,14,15].

Unlike lipidome alterations, fluoxetine-induced gene expression differences detected in our study were subtle. Still, the expression of lipid-related enzymes was slightly, but significantly more affected by the fluoxetine administration than the average gene expression. Furthermore, our results match multiple reports demonstrating the fluoxetine-dependent increase in expression of synapse-related genes [70,71,72,73], and the immunomodulatory and anti-inflammatory effects of the drug [74,75,76,77]. Correspondingly, fluoxetine-associated gene expression differences represent an inversion of gene expression alterations associated with psychiatric disorders including schizophrenia, bipolar depression, and unipolar depression. Specifically, numerous studies showed disorder-associated inflammation increase [78,79,80,81,82,83] and anti-inflammatory effects of commonly used medication [76,84], as well as a decrease in expression of synaptic genes in the brains of depressive patients [85,86] and patients with schizophrenia [87].

In summary, our study represents an effort to systemically assess, at three levels of the molecular phenotype, the long-term effects of the commonly used antidepressant fluoxetine on the developing primate brain. We report subtle fluoxetine-associated gene expression alterations representing a reversal of commonly reported pathological gene expression changes. However, we detect main fluoxetine-associated effects at the brain lipidome composition level, centering around the decrease in PUFA content. Combined with the reported link between PUFA deprivation and psychiatric impairments, these observations point towards the general alterations of fatty acid metabolism as a potential source of adverse fluoxetine treatment effects.

## 4. Materials and Methods

### 4.1. Assurance of Compliance with Animal Codes

All procedures during the in vivo phase of the study followed the Guide for the Care and Use of Laboratory Animals of the National Research Council. The California National Primate Research Center (CNPRC) is accredited by the Association for Assessment and Accreditation of Laboratory Animal Care. Protocols for this project were approved before implementation by the UC Davis Institutional Animal Care and Use Committee. An international sample shipping was conducted according to CITES guidelines.

### 4.2. Subjects and Design

Thirty-two male rhesus macaques, one year of age, were selected from the outdoor colony at CNPRC on the basis of health history and behavioral screening as described previously [28]. They were transferred to pair-housing in indoor caging and assigned to treatment groups (fluoxetine, vehicle, *n* = 16/group) randomly, balancing for animal size, age, cage location, and *MAOA* polymorphism genotype. At the conclusion of the study, brain tissue was made available for multi-omics assays of 11 control animals and 10 fluoxetine-treated animals.

### 4.3. Fluoxetine Dosing

Daily drug treatment with fluoxetine was initiated at one year of age and continued for two years until animals reached three years of age. Fluoxetine (Webster Veterinary Supply, Devens, MA, USA) was mixed with flavored syrup or liquefied baby food and delivered via oral syringe directly into the mouth. Monkeys were trained to come forward and place the tip of the syringe in their mouths to initiate dosing. Controls received only the flavored vehicle.

The fluoxetine dose resulted in serum levels seen in children treated clinically with fluoxetine. It was based on preliminary pharmacokinetic studies [88], as well as previous experience with this drug in macaques [17,89,90,91,92,93,94]. For the first 11 months 1.6 mg/kg was administered daily. This allowed training for consistent compliance with dosing and induction of metabolizing enzymes. After analysis of steady state serum levels, the dose was adjusted to 2.4 mg/kg for the remainder of the dosing period followed by a four-week tapered discontinuation period and a two-week washout at the onset of the post-dosing period.

### 4.4. MAOA Genotyping

Most three-month old rhesus monkeys at CNPRC are genotyped for *MAOA* polymorphisms and this information is available in their electronic record. VNTR polymorphism (*rhMAOA-LPR*) genotyping was performed by the Veterinary Diagnostic Laboratory using PCR with forward and reverse primers. VNTR repeat lengths of 5 and 6 were classified as “hi-MAOA” while repeat length 7 was classified as “low-MAOA” based on 26% higher transcription rate for the 5 and 6 repeats [69]. Allele frequencies in rhesus have been estimated at 60% “hi-MAOA” and 40% “low-MAOA” [29,69].

### 4.5. Necropsy and Medial Prefrontal Cortex Tissue Collection

At the conclusion of the study, at 4 years of age, PET scans for serotonin binding potential were conducted, and necropsies were performed to obtain brain samples for evaluation of residual effects of fluoxetine on brain one year after discontinuation of dosing. Monkeys were anesthetized with ketamine (10 mg/kg i.m.) followed by an overdose of pentobarbital (120 mg/kg i.v.). Animals were then perfused with 1 L of warm heparinized saline followed by 3 L of cold heparinized saline and a flush with 500 mL cold saline. Afterwards, brains were quickly removed and put in a bag of cold saline surrounded with wet ice for 10 min. Brains were placed in a custom made 5 mm thick high density polyethylene plexiglass mold, cut into coronal sections and 5 mm slices placed on a wet ice chilled aluminum surface. Slices were cut into right and left halves along the midsagittal line and foil was placed between the slabs to keep them separated but in the proper orientation. PLC samples (6–8 punches) were collected using a 1 mm diameter punching needle (Leica Biosystems #39443001RM). Punches were taken from the cortex of area 6/32 forming the dorsal bank of the cingulate sulcus from slices extending over frontal planes, Bregma 8.00 to −7.50 mm, according to the stereotaxic atlas of rhesus monkey brain [95]. For punching, the frozen slices were placed on a glass plate chilled with dry ice. All punches were immediately transferred to a cryovial, frozen in liquid nitrogen and stored at −80 °C. The frontal cortex was selected for the multi-omics studies because serotonin binding in this area differed between fluoxetine-treated animals and controls during the post-dosing period [34]. The medial prefrontal cortex in particular is important in top-down control of brain serotonin systems [96]. Immediate freezing in liquid nitrogen and storage at −80 °C is one of the possible protocols recommended for ensuring lipid stability during sample preparation and storage [97].

### 4.6. Transcriptomics

Gene expression analysis was carried out by IMGM Laboratories GmbH, Martinsried, Germany. Total RNA was isolated from about 30 mg macaque brain tissue in two batches using the RNeasy Lipid Tissue Mini Kit (Qiagen) according to the manufacturer’s instructions including on-column DNase digestion. Total RNA was eluted in 30 µL RNase-free water. An aliquot of each isolated total RNA sample was used to determine RNA concentration and purity on the NanoDrop ND-1000 spectral photometer (Peqlab). All samples were analyzed on the 2100 Bioanalyzer (Agilent Technologies, Waldbronn, Germany) using RNA 6000 Nano LabChip Kits (Agilent Technologies, Waldbronn, Germany). The RNA sequencing library was prepared from the isolated RNA samples with the Illumina TruSeq^®^ Stranded mRNA HT technology using fragmentation, a poly-T oligo pulldown and sequencing adapter ligation. RNA sequencing was performed on the Illumina NextSeq^®^ 500 next generation sequencing system and its high output mode with 1 × 75 bp single-end read chemistry. The resulting reads were quality controlled and mapped against the *Macaca mulatta* reference genome (Mmul_8.0.1/rheMac8) using the CLC Genomics Workbench (QIAGEN) with the following parameters: mismatch cost = 2, insertion/deletion cost = 3, length fraction = 0.8, similarity fraction = 0.8, maximum number of hits per read = 5.

Genes with median RPKM (Reads Per Kilobase of transcript, per Million mapped) values < 2 or containing missing values were removed from the downstream analysis. Next, only protein-coding genes, according to biomaRt package [98], were considered for analysis. All gene IDs were converted from gene symbols to ENTREZ IDs using clusterProfiler and org.Mmu.eg packages [99,100]. The RPKM values of the remaining genes were log2-transformed and upper-quartile normalized.

Enzymes directly linked to lipid compounds were defined in the following way, using KEGG API and KEGG LinkDB [101]. Lipid classes quantified in the lipid dataset were converted to the corresponding LIPID MAPS classes, defined by the first 8 symbols of the LIPID MAPS ID [102]. Next, LIPID MAPS IDs corresponding to these classes (LIPID MAPS entries with the corresponding first 8 symbols) were linked to KEGG compound IDs. Links not present in KEGG database were manually added: LMGP0102 C05212, LMGP0103 C00958, LMGP0203 C04756, LMGL0201 C00165, LMGL0101 C02112, LMFA0101/LMFA0103 C00638, LMGP0202 C04475 (Table S11). Further, KEGG compounds were linked to KEGG reactions, and then KEGG reactions to KEGG enzymes, producing in the end a list of enzymes directly linked to the lipid classes quantified in the lipid dataset (Table S9).

### 4.7. Sample Preparation

All sample preparation steps were performed on ice. Hydrophilic metabolites and lipophilic compounds were isolated by liquid/liquid separation between methyl tert-butyl ether (MTBE) (HPLC quality, Sigma–Aldrich GmbH, Germany) and water [103]. For this purpose, 5 mg of frozen sample was transferred into beat tubes (MN Bead Tubes Type B, Macherey-Nagel, Germany), 200 µL ice old methanol (LC-MS quality, Honeywell, Germany) was added, and the sample was milled in a cooled tissue lyser (−5 °C, Precellys, bertin instruments, France) under permanent nitrogen flow. Afterwards, 300 µL MTBE was added and extraction was performed in a sonic bath for 30 min on ice. After addition of 400 µL water to induce phase separation, samples were held in a sonic bath for 15 min.

After centrifugation (14,000 rpm, 4 °C, 15 min), upper phase was taken and solvent was evaporated to dryness with a Speed Vac concentrator at room temperature. Pellets were reconstituted in 100 µL of acetonitrile:isopropanol (7:3, *v/v*) mixture and then diluted two times prior to liquid chromatography mass spectrometry measurement in positive mode with acetonitrile:isopropanol (7:3, *v/v*). Measurement in negative polarity was carried out without dilution. 

150 mL MTBE was added to the lower phase (15 min, sonic bath). Following centrifugation (14,000 rpm, 4 °C, 15 min), the upper phase was removed and the hydrophilic fractions were diluted 1:1 with methanol. Following centrifugation (14,000 rpm, 4 °C, 15 min), the upper phase was removed and the hydrophilic fractions were diluted 1:1 with methanol.

### 4.8. Metabolomics

Ultrahigh resolution mass spectra were acquired on a 12 T solariX FT-ICR mass spectrometer (Bruker Daltonics, Billerica, MA, USA) equipped with an Apollo II electrospray source (Bruker Daltonics). Broad band detection mode with a time domain transient of 2 Megaword was applied. The instrument was calibrated with a 1 ppm arginine solution resulting in a mass error below 100 ppb. Extracts were injected with 120 µ/h, 350 scans were acquired for positive electrospray mode. Mass lists were generated with a signal-to-noise ratio (S/N) of four, exported, and combined into one data matrix by applying a 1 ppm window.

Resulting features were filtered according to the number of missing values, and features with more than 10% missing values across samples were removed from downstream analysis. The missing values were replaced by the minimum value across the whole data table. Resulting abundances were log2-transformed and upper-quartile normalized. Metabolite features were annotated based on *m/z* values matched to KEGG database [101] with 1 ppm mass accuracy cutoff (Table S5).

### 4.9. Lipidomics

The liquid chromatography/mass spectrometry analysis was performed on a Waters Acquity I-class UPLC system (Waters, Manchester, UK) and a Q Exactive Orbitrap mass spectrometer (Thermo Fisher Scientific, USA) equipped with a heated electrospray ionization probe. Chromatographic separation of lipids was achieved by using a reverse phase ACQUITY UPLC BEH C8 column (2.1 × 100 mm, 1.7 μm, Waters Co., Milford, MA, USA) equipped with Vanguard precolumn at a flow rate of 0.4 mL/min and temperature kept on 60 °C. The mobile phases were composed of 0.1% formic acid in water containing 10 mM ammonium acetate (Buffer A), and 0.1 % formic acid in a mixture of acetonitrile and isopropanol (7:3) containing 10 mM ammonium acetate (Buffer B). Separation was carried out by gradient elution according to the following profile: 1 min 55% B, 3 min linear gradient from 55% to 80% B, 8 min linear gradient from 80% B to 85% B, and 3 min linear gradient from 85% B to 100% B. After 4.5 min washing with 100% B the column was re-equilibrated with 55% B for 4.5 min. The injection volume was set at 3 μL. Mass spectra were acquired in full-scan mode within *m/z* range 100 to 1500 in profile mode for positive and negative polarities. The resolution was set as 70,000 (at *m/z* 200), the AGC target at 1 × 10^6^, max IT at 100 ms. Ion source was operated with following parameters: capillary temperature, 250 °C; aux gas heater temperature, 350 °C; spray voltage, (±)4.5 kV; S-lens RF level, 70; sheath gas (N_2_) flow rate, 45 arbitrary units (a.u.); auxiliary gas (N2) flow rate, 20 a.u.; sweep gas (N2) flow rate, 4 a.u.

All samples were randomized before injection and the QC samples (pooled mixture of 10 µL aliquots of all the samples after their extraction and dilution) were inserted at every 12th position throughout the batch to estimate technical variability of retention times, mass accuracy and signal drift. Blank solvent samples (solvent used for pellets reconstitution) were injected several times at the end of the batch and were later used to eliminate system contamination. Acetonitrile (LC-MS grade), isopropanol (LC-MS grade), and water (LC-MS grade) were obtained from Scharlab, S.L. (Barcelona, Spain). Formic acid used in the mobile phases was HPLC-MS grade (98–100%) and obtained from LIChropur (Darmstadt, Germany). Ammonium acetate was HPLC grade (≥99%) and obtained from Sigma-Aldrich (St. Louis, MO, USA). Additionally, QC samples with added internal standards (SPLASH LIPIDOMIX Mass Spec Standard, Avanti Polar Lipids) were injected at the end of the batch to allow for retention time correction between the current dataset and an in-house database of MS/MS validated lipid species. Internal standards were not added to the control and treatment samples, and absolute concentrations are not reported in this study.

For fragmentation analysis, several QC injections were made at the end of the positive and negative polarity batches. Data was recorded in data-dependent (DDA) acquisition mode. Ion source operation parameters were kept the same as for the main batch (full scan mode). DDA settings: full-scan mode was acquired with resolution of 70,000, AGC target: 5 × 10^5^, IT: 50 ms. The fragmentation mode was operated with resolution of 17,500 at *m/z* 200, AGC target: 2 × 10^4^, IT: 100 ms, mass isolation window: 1.2 Da, stepped normalized collision energy: 15, 25, 30%, dynamic exclusion 12 s, and intensity threshold of 8 × 10^3^. Top 10 intense ions were selected for fragmentation. The spectra were recorded in the profile mode.

MS1 spectra were processed using XCMS software [104], with “centWave” method for peak detection, “obiwarp” method for retention time correction, and “fillPeaks” method for missing value imputation (for more details see Supplementary Methods). Duplicated features were deleted. Features representing clusters of co-eluting derivative products of the same compound were removed. Isotopes were removed as well. For more details on these procedures see Supplementary Methods. Abundance values were log2 transformed. Missing values as reported by XCMS were replaced by random values sampled from a normal distribution with mean 12 and standard deviation 0.5, which corresponds to the approximate noise level seen in our experimental method. Features with values ≤13 (mean + 2std of random values used to fill the missing values) in ≥25% of samples were removed from the downstream analysis. Contaminants were filtered out using solvent blank samples according to the following rule: features with mean abundance in samples < mean abundance in blank samples + 3 (in log2 scale) were removed from the analysis. Features with high technical variability were removed using QC samples according to the following rule: features with standard deviation across QC samples > 0.25 (in log2 scale) were removed from the analysis. For the features retained after above-described procedures, the resulting abundances across samples were upper-quartile normalized. 

Lipids were putatively annotated by *m/z* values and retention times. For each considered lipid class, lipid features were matched by *m/z* values with mass accuracy cutoff of ppm = 10 to a generated theoretical *m/z* list, and then matches with inappropriate retention times were deleted as false annotations. Appropriate retention times were determined based on in-house retention time for previously annotated lipid species, as well as chain length and double bond content of the presumed annotations. For more information, see Supplementary Methods. The lipid species detected in our analysis were reported with one adduct for each lipid class (Table S6). The lipid classes considered during the annotation phase, but not detected consisted of phosphatidylserine (PS), phosphatidylglycerol (PG), phosphatidic acid (PA), as well as sphingolipids with a different number of total hydroxyl groups than reported in Table S6 (SHexCer;O1, Cer;O1, Cer;O3, SM;O1, SM;O3).

To confirm putative annotations according to their MS/MS fragmentation patterns, we used LipidHunter [105] with MS level tolerance of ±5 ppm, MS/MS level tolerance of 10 ppm, MS level intensity threshold of 1000, MS/MS level intensity threshold of 10, isotope score ≥ 80, rank score ≥ 49. Of the 373 putatively annotated lipid features, 78 (21%) generated informative MS/MS fragmentation patterns and were annotated by LipidHunter. All 78 LipidHunter annotations matched their corresponding putative annotations. Of note, lipids putatively annotated as free fatty acids were not covered by the fragmentation procedure, based on the limited applicability of LC-MS/MS protocol to this lipid class.

### 4.10. Statistical Analysis

To visualize variation in lipid, metabolite, and gene expression abundance across samples, we used multi-dimensional scaling with Pearson correlation used as a metric of sample similarity.

To assess the effect of fluoxetine, we performed Welch *t*-test with unequal variance assumption and Benjamini-Hochberg (BH) correction for multiple testing. False discover rate (FDR) 10% was used to define the treatment-associated lipids. To assess the overall effect of fluoxetine on lipid abundances, we performed the following permutation test. After 1000 permutations of sample labels, we performed the same *t*-test procedure followed by the BH correction described above. The permutation *p*-value was defined as the fraction of permutations for which the number of lipids passing the same 10% FDR cutoff was equal or greater than the number of treatment-associated lipids.

For group-based analysis assessing the significance of the treatment effect at the level of individual lipid classes, we used Gene Set Enrichment Analysis (GSEA) algorithm (R package “fgsea” [106]) with lipid classes in place of gene sets. T-test statistic was used to sort the lipids for this GSEA (Table S6). We report normalized enrichment score (NES) and adjusted p-values according to the package output.

To calculate the average effect of fluoxetine depending on the degree of unsaturation, we considered glycero- and glycerophospho- lipid classes, specifically, MAG, DAG, TAG, PC, PE, PC-O, PC-P, PE-O, PI, LPC, LPE, and FFA. The remaining classes (sphingolipids) usually do not contain high amounts of PUFAs and were not considered in this analysis. The chosen classes were separated into three groups based on the number of fatty acid residues. For each of these groups, the log2 fold-change of fluoxetine-treated macaques versus control was averaged for all the lipids with the same amount of double bonds, according to the lipid annotation.

To assess the interaction of *MAOA* genotype and treatment, we first performed ANOVA with terms treatment, genotype, and treatment-by-genotype. Next, we performed hypergeometric test to assess over-representation of lipid features with nominal *p*-value < 0.05 for treatment-by-genotype in the set of treatment-associated lipids, as well as different lipid classes.

To assess the effect of fluoxetine on lipid enzymes, we compared median log2 fold-change of all lipid enzymes as defined in section “Gene expression data pre-processing” to the median log2 fold-change for 10,000 random subsets of genes of the same size. Permutation *p*-value was defined as the fraction of these subsets for which the median log2 fold-change was equal or higher than the median log2 fold-change for lipid enzymes.

MetaboAnalyst package [107] was used to search for metabolite groups significantly enriched in treatment-associated metabolites, defined as annotated metabolites with nominal Welch *t*-test *p*-value < 0.1 for fluoxetine-treated macaques compared to control (Table S5).

For group-based analysis assessing the significance of the treatment effect at the gene expression level, we used GSEA algorithm (“clusterProfiler” R package [99]) with 1,000,000 permutations and gene ontology (GO) to define gene sets. Log2 fold-changes were used to sort the genes for the GSEA (Table S7).

## Data Availability

The tables for gene expression, metabolite abundances, and lipid abundances are included in the Supplementary Materials. Datasets not included in the supplementary material are available from the corresponding author upon reasonable request.

## Supplementary Materials

The following are available online at https://www.mdpi.com/article/10.3390/ijms22158089/s1, Table S1: Sample information. Table S2: Gene expression table. Table S3: Table of polar metabolite abundances. Table S4: Table of lipid abundances. Table S5: Polar metabolite information, including annotation and *p*-values for *t*-test of fluoxetine vs control groups. Table S6: Lipid information, including annotation and *p*-values for *t*-test of fluoxetine vs control groups. Table S7: Gene information, including *p*-values for *t*-test of fluoxetine vs control groups. Table S8: Results for lipid class analysis (Gene Set Enrichment Analysis method). Table S9: Table of lipid enzymes corresponding to the lipid classes. Table S10: Gene Set Enrichment Analysis (GSEA) results for gene expression. Table S11: Table of links from KEGG compounds to LMSD identifiers.

## Abbreviations

AA	arachidonic acid
Cer	ceramide
DAG	diacylglycerol
DHA	docosahexaenoic acid
FFA	free fatty acid
HexCer	hexosylceramide
LPC	lysophosphatidylcholine
LPE	lysophosphatidylethanolamine
MAG	monoacylglycerol
MAOA	monoamine oxidase A
PC	phosphatidylcholine
PC-O	plasmanylphosphatidylcholine
PC-P	plasmenylphosphatidylcholine
PE	phosphatidylethanolamine
PE-O	plasmanylphosphatidylethanolamine
PE-P	plasmenylphosphatidylethanolamine
PI	phosphatidylinositol
PUFA	polyunsaturated fatty acid
SHexCer	sulfatide
SM	sphingomyelin
TAG	triacylglycerol
;O1	total number of hydroxyl groups = 1 (sphingolipids)
;O2	total number of hydroxyl groups = 2 (sphingolipids)
;O3	total number of hydroxyl groups = 3 (sphingolipids)
