# Supplementary material for: Long-Term Fluoxetine Administration Causes Substantial Lipidome Alteration of the Juvenile Macaque Brain
Source: Int J Mol Sci. 2021 Jul 28;22(15):8089. doi: 10.3390/ijms22158089 (PMC8348031; doi:10.3390/ijms22158089)
Supplement: Supplementary file 1 [file ijms-22-08089-s001.zip › prozac_SupplFigures_v8.pdf]

Long-term fluoxetine administration causes substantial lipidome alteration of the juvenile macaque brain

Anna Tkachev[1,2,§], Elena Stekolshchikova[1,§], Daniil M. Bobrovskiy[3], Nickolay Anikanov[1], Polina Ogurtsova[1], Dong Ik Park[4,#], Anja K.E. Horn[5], Daria Petrova[1], Ekaterina Khrameeva[2,*], Mari S. Golub[6,*], Christoph W. Turck[4,*], Philipp Khaitovich[1,*]

[1] Center for Neurobiology and Brain Restoration, Skolkovo Institute of Science and Technology, Moscow, 121205, Russia
[2] Center of Life Sciences, Skolkovo Institute of Science and Technology, Moscow, 121205, Russia
[3] Faculty of Bioengineering and Bioinformatics, Moscow State University, Moscow 119234, Russia
[4] Max Planck Institute of Psychiatry, Munich, Germany
[5] Ludwig Maximilians University, Department I, Institute of Anatomy and Cell Biology, Munich, Germany
[6] California National Primate Research Center, University of California, Davis, CA, USA

[§] Contributed equally

[*] Correspondence should be addressed to: e.khrameeva@skoltech.ru, msgolub@ucdavis.edu, turck@psych.mpg.de, and khaitovich@eva.mpg.de

[#] Present address:

Aarhus University, Department of Biomedicine, Ole Worms Allé 3, 8000 Aarhus C, Denmark

Supplementary Figures

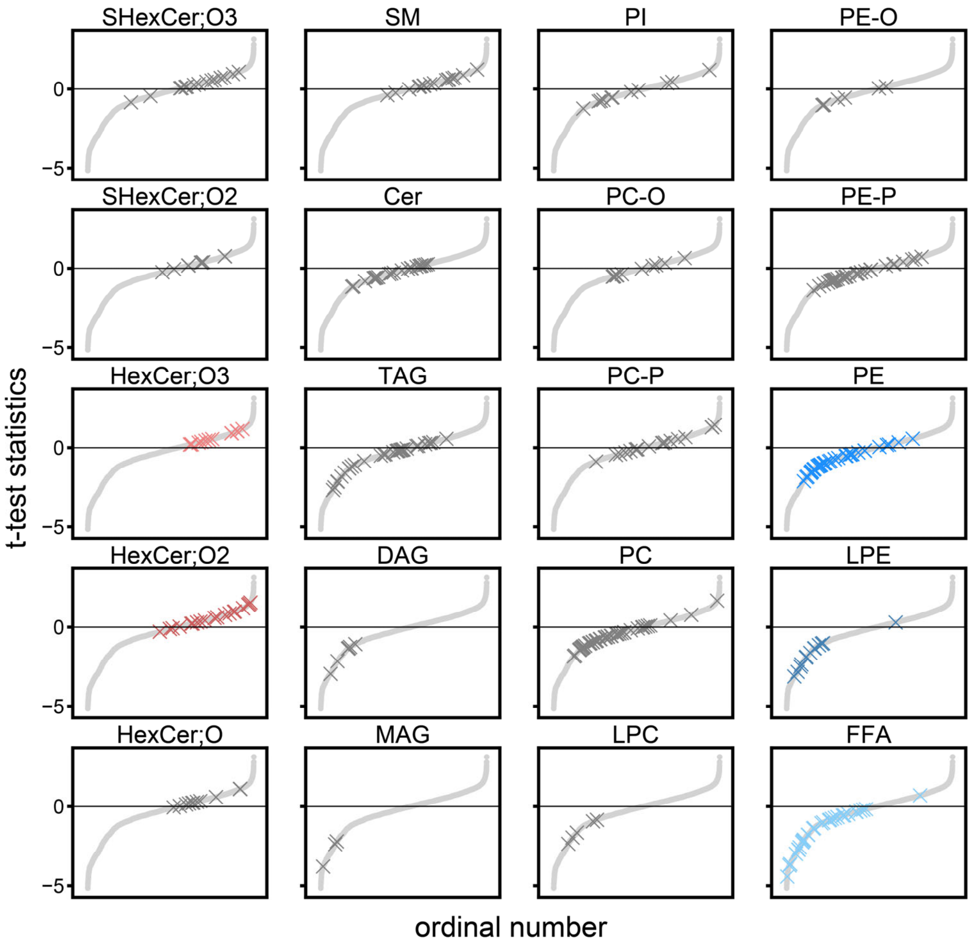

Supplementary Figure S1

T-test statistic values for each lipid class. Grey lines: t-test statistics for all lipids, ordered from smallest to largest value, plotted against the lipids' corresponding ordinal values. 'X' marker points: the ordered t-test statistics and corresponding ordinal values for lipids from each lipid class, marked on the top. Colors indicate lipid classes demonstrating significant enrichment in the treatment-control differences and are the same as colors in Figure 2A.

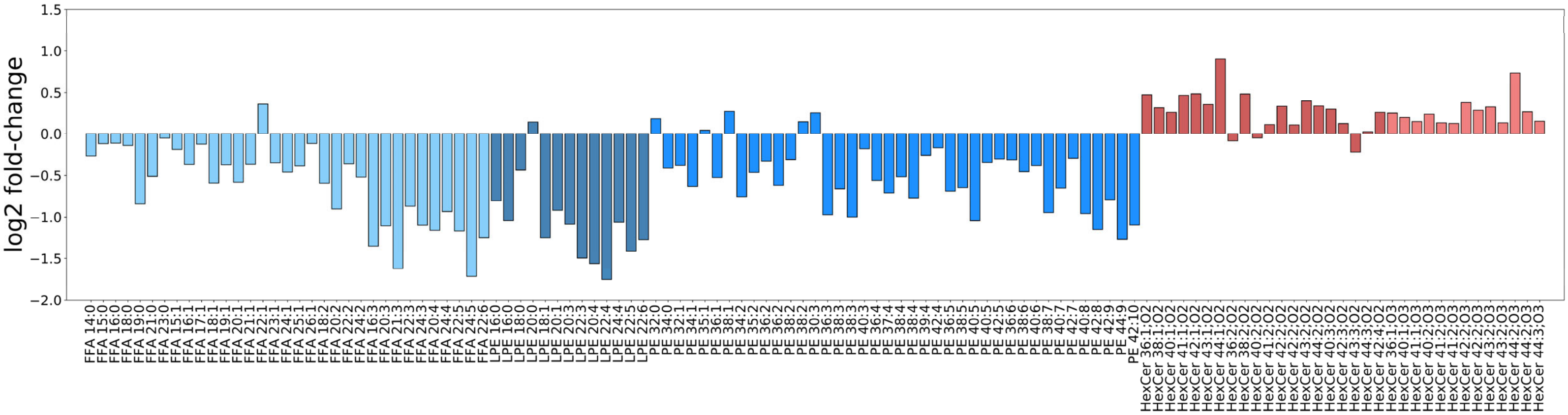

Supplementary Figure S2

Base two log-transformed fold change (log2 FC) values calculated between treated and control animals for lipids in FFA, LPE, PE, HexCer;O2, and HexCer;O3 lipid classes.

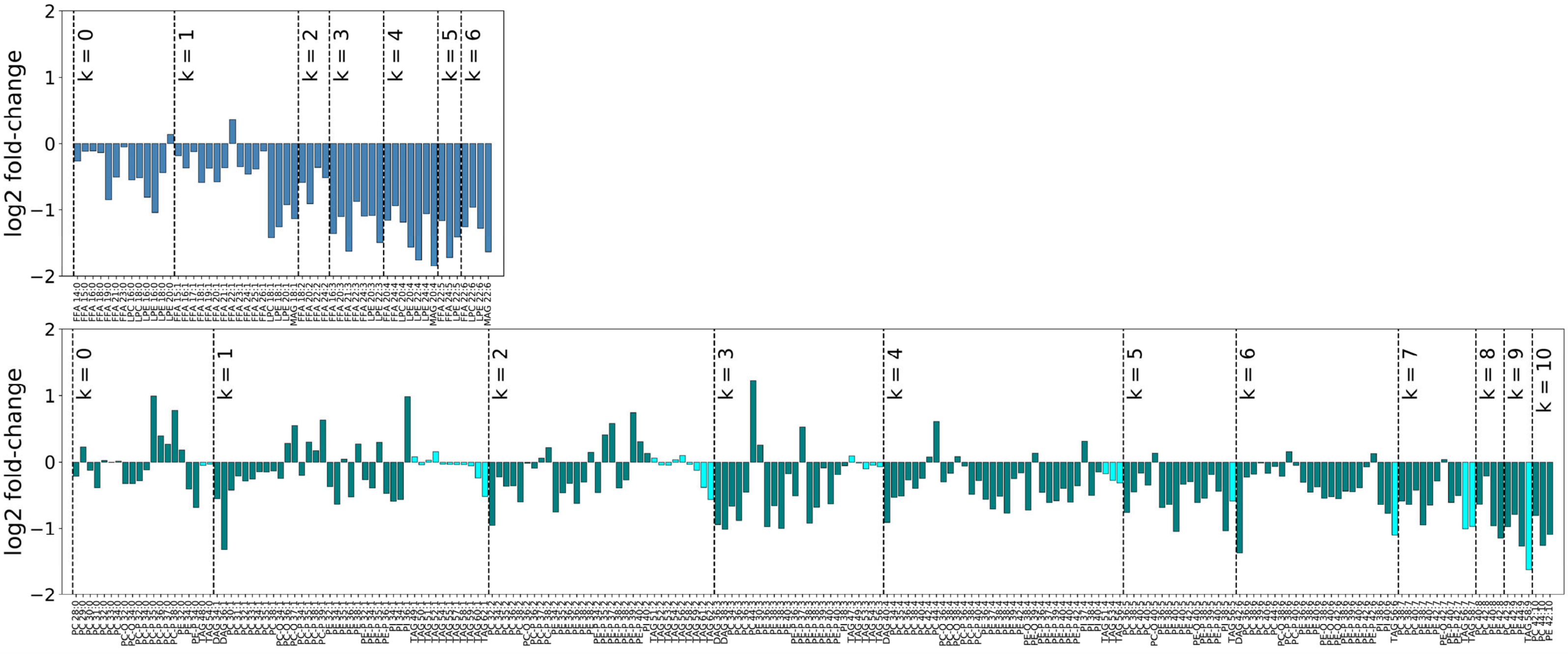

Supplementary Figure S3

Base two log-transformed fold change (log2 FC) values calculated between treated and control animals for lipids containing one (top, dark blue), two (bottom, green) and three (bottom, light blue) fatty acid residues, excluding sphingolipids. Lipids are ordered by increasing unsaturation. Dashed lines demarcate lipids with the same number of double bonds (k).

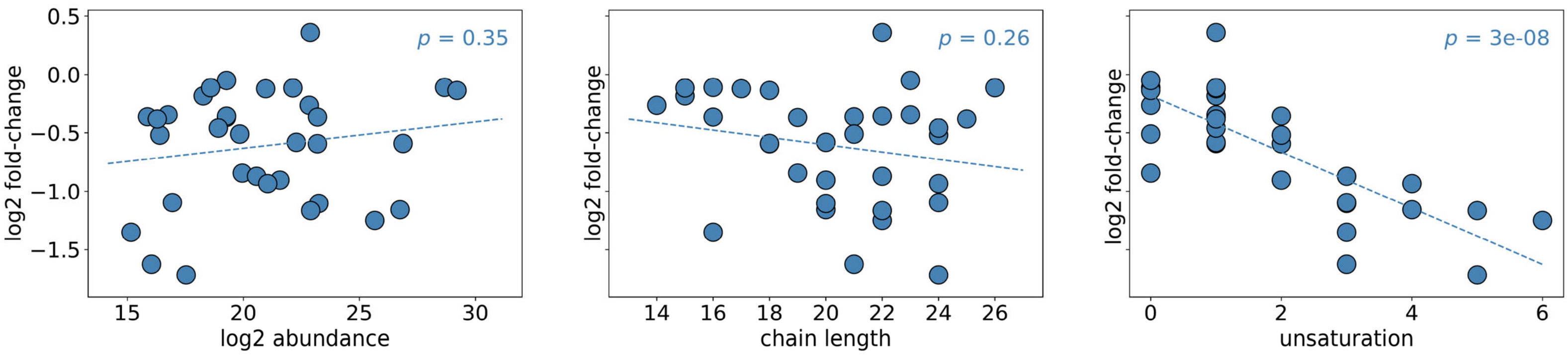

Supplementary Figure S4

Base two log-transformed fold change (log2 FC) values calculated between treated and control animals for lipids in free fatty acid class (FFA) depending on abundance (left), chain length (middle) and level of unsaturation (right). Dashed lines indicate linear regression lines fitted to each group, corresponding p-values are indicated in the top right corner.

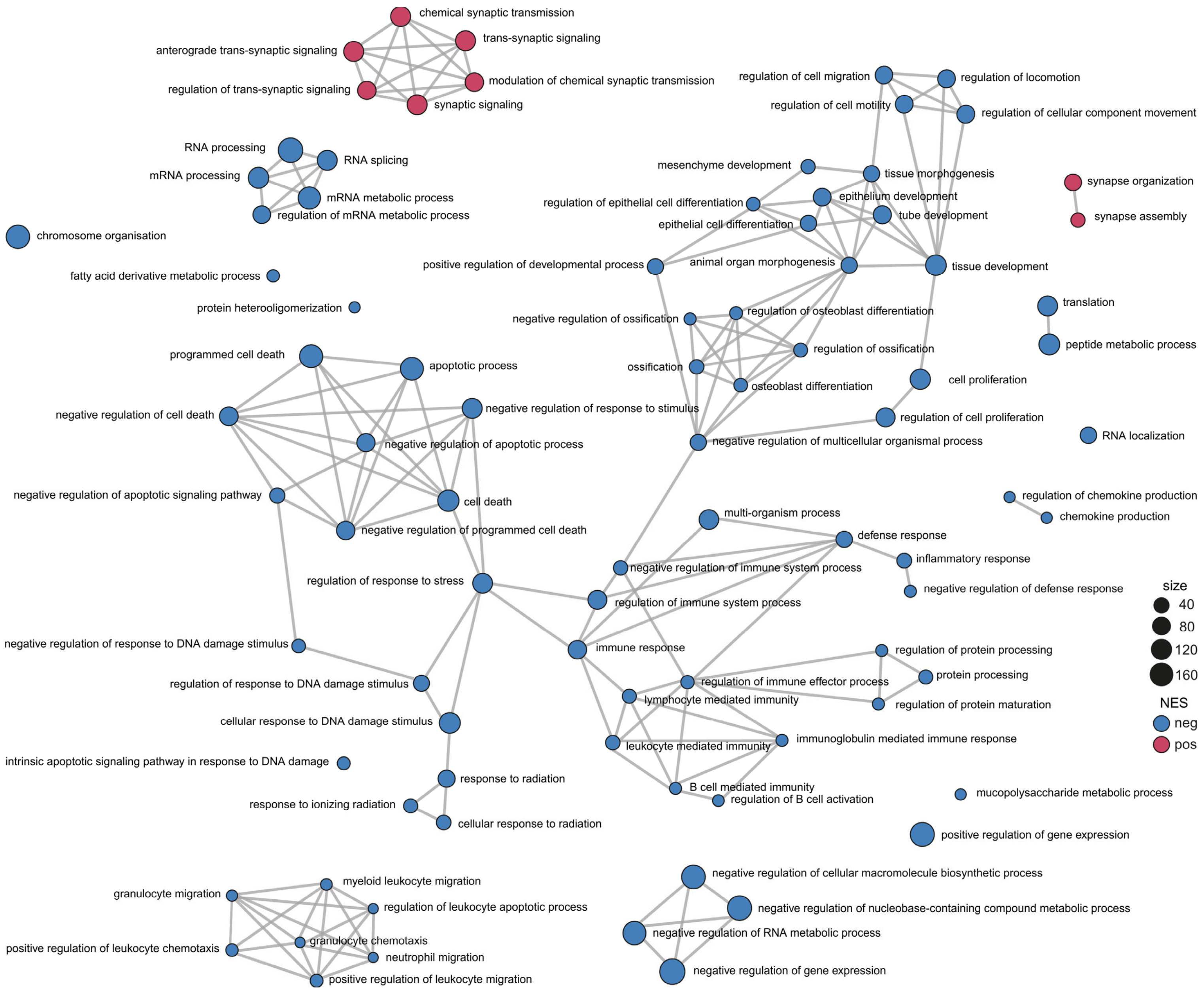

Supplementary Figure S5

Gene ontology (GO) terms enriched in gene expression differences between treated and control animals (GSEA, adjusted $p < 0.05$). One point indicates one GO term. Colors show negative (blue) and positive (red) normalized enrichment score values, which correspond to the dominant direction of expression change between treated and control animals within the GO term. Symbol size is proportional to the number of genes in the GO term. Edges connect functionally related GO terms.
