# Supplementary material for: Long-Term Fluoxetine Administration Causes Substantial Lipidome Alteration of the Juvenile Macaque Brain
Source: Int J Mol Sci. 2021 Jul 28;22(15):8089. doi: 10.3390/ijms22158089 (PMC8348031; doi:10.3390/ijms22158089)
Supplement: Supplementary file 1 [file ijms-22-08089-s001.zip › prozac_SupplMethods_v8.pdf]

Long-term fluoxetine administration causes substantial lipidome alteration of the juvenile macaque brain

Anna Tkachev[1,2,§], Elena Stekolshchikova[1,§], Daniil M. Bobrovskiy[3], Nickolay Anikanov[1], Polina Ogurtsova[1], Dong Ik Park[4,#], Anja K.E. Horn[5], Daria Petrova[1], Ekaterina Khrameeva[2,*], Mari S. Golub[6,*], Christoph W. Turck[4,*], Philipp Khaitovich[1,*]

[1] Center for Neurobiology and Brain Restoration, Skolkovo Institute of Science and Technology, Moscow, 121205, Russia
[2] Center of Life Sciences, Skolkovo Institute of Science and Technology, Moscow, 121205, Russia
[3] Faculty of Bioengineering and Bioinformatics, Moscow State University, Moscow 119234, Russia
[4] Max Planck Institute of Psychiatry, Munich, Germany
[5] Ludwig Maximilians University, Department I, Institute of Anatomy and Cell Biology, Munich, Germany
[6] California National Primate Research Center, University of California, Davis, CA, USA

[§] Contributed equally

[*] Correspondence should be addressed to: e.khrameeva@skoltech.ru, msgolub@ucdavis.edu, turck@psych.mpg.de, and khaitovich@eva.mpg.de

[#] Present address:

Aarhus University, Department of Biomedicine, Ole Worms Allé 3, 8000 Aarhus C, Denmark

Supplementary Methods

XCMS parameters

The following XCMS parameters and methods were used for data pre-processing:

xset <- xcmsSet (method = "centWave", peakwidth = c(10, 30), ppm = 15, noise = 50, snthresh = 3, mzdiff = 0.003, prefilter = c(3, 100), mzCenterFun = "wMean", integrate = 1, fitgauss = FALSE, verbose.columns = FALSE

xset <- retcor (xset, method = "obiwarp", plottype = "none", distFunc = "cor_opt", profStep = 0.6352, center = 2, response = 1, gapInit = 0.448, gapExtend = 2.4, factorDiag = 2, factorGap = 1, localAlignment = 0

xset <- group (xset, method = "density", bw = 3, mzwid = 0.008, minfrac = 0.05, minsamp = 10, max = 50)

xset <- fillPeaks(xset)
groups (xset,method="maxint",value="into")

Duplicated peaks

XCMS peak picking parameters that are not ideal for a given peak shape can result in one lipid signal being split into two peaks in the resulting feature table, identifiable as two highly correlated features with almost identical m/z values and retention time values. We dealt with this issue by grouping all features with pairwise difference in m/z values < 10 ppm and retention time difference < 0.05 minutes, keeping only one feature from each group.

Co-eluting peak clusters

Untargeted LCMS lipidomics experiments can produce large clusters of lipid features that are derived from the same lipid compound, identifiable as a large number of well-correlated lipid features with identical retention times. We dealt with this issue by deleting larger-than-average groups of lipid features that fall into a small retention time window as follows. First, we defined a window of 0.005 minutes and calculated the number of lipid features with retention times falling into this window, depending on the position of the window across the retention time gradient ("sliding histogram"). Next, we used kernel density estimation (python package KernelDensity, kernel='gaussian', bandwidth=0.5; this calculates the logarithm of the density) on the sliding histogram values, and calculated the smoothed (or averaged) sliding histogram by taking the exponent of the kernel density estimation and normalizing it by the area under the sliding histogram. We defined a first cutoff function as this smoothed histogram. Next, we defined a spike in the sliding histogram as a group of adjacent values of the sliding histogram that are larger than the cutoff function. Each spike corresponds to a retention time boundary, and a set of lipid features that fall into this retention time boundary. The height of the spike is the maximum value of the sliding histogram in this retention time boundary. We aimed to delete abnormal spikes that correspond to co-eluting peak clusters. Because we expected co-eluting peak clusters to be a derivative from one lipid compound (which will have the highest intensity), we expected most peaks in a co-eluting cluster to have m/z values higher than this original lipid compound. However, this might not be true for all co-eluting peak clusters. For this reason, we defined two types of abnormal spikes. The first type of abnormal spikes was defined as spikes with height > 40. For the second type of abnormal spikes, we defined a second cutoff function as 4*smoothed histogram +10. We defined as abnormal any spike for which (1.) the height was larger than this cutoff function, and

for which (2.) the percent of lipid features in this spike with m/z values > m/z value of the lipid feature with highest intensity in this spike was larger than 75%. We deleted from downstream analysis all lipid features that corresponded to an abnormal spike.

Isotope deletion

Isotopes were grouped based on differences in m/z and retention times of the lipid features. Additionally, we used pairwise correlations across samples, because we expect variability in isotope intensities to be explained only by unbiased random error in measurements (any biological variability or technical biases will be identically reflected in isotopes), and we expect these correlations to be quite high.
First, lipid features were separated into isotope pre-groups. Differences between m/z values (m/z_diff) and differences in retention times (rt_diff) were calculated for each pair of lipid features, and features with m/z_diff<0.01 and rt_diff< 10 seconds were considered to be connected. Next, we located all the connected components, each connected component defining an isotope pre-group. Because two features in a pre-group might be connected through a chain of connected pairs, the resulting m/z_diff and rt_diff could be much higher than the above-defined thresholds, and one pre-group could contain multiple isotope groups.
Next, for each pre-group we defined the isotope groups. Starting from lipid feature X with highest intensity in the pre-group, we identified the lipid features Y satisfying the following criteria: 1000000*(m/z of X - m/z of Y + C13 - C12)/ (m/z of X) < 10, difference in retention time < 1.5 seconds, correlation across samples < 0.8, and intensity of Y < intensity of X. If exactly one feature was found, this feature was added to the isotope group and the procedure was continued recursively. If no feature or multiple features were found, the procedure was stopped and the lipid features Y were not added to the isotope group. In all cases, lipid features Y were deleted from the pre-group, as well as feature X. Once the procedure stopped, if there were lipid features remaining in the pre-group, the algorithm was repeated recursively, starting with the remaining lipid feature with highest intensity.
This algorithm defined isotope groups. For each isotope group, only the highest intensity feature was retained in the data table, and all other lipid features (their higher level isotopes) were deleted from analysis.

TL annotation

We used a semi-automated procedure based on m/z values and retention times for the putative annotation. For each considered lipid class, we generated a theoretical table of neutral masses corresponding to the lipid species with the various number of double bonds and chain lengths. Each lipid class was annotated in one ionization mode using one adduct of choice (Table 6). The lipid classes we reported in the data were: MAG, DAG, TAG, FFA, PC, LPC, PC-O, PC-P, PE, LPE, PE-O, PE-P, PI, Cer, SM, HexCer;O1, HexCer;O2, HexCer;O3, SHexCerO2; SHexCerO3. The lipid classes we considered, but did not discover in the data were: PS, PG, PA, Cer;O1, Cer;O3, SM;O1, SM;O3, SHexCer;O1.
Starting from a lipid class and its corresponding adduct, we matched the theoretical m/z to the m/z values of the lipid features with a mass accuracy threshold of 10 ppm. Next, a model for the retention times of the lipid species was built, and only lipid species fitting into this model were retained in the putative annotation, according to the following procedure. We started with an in-house database of retention times of verified lipid species annotation, and retention time alignment between the current retention times and the database retention times based on linear interpolation between retention times of the internal standards. The retention times of the in-house database were matched to the current retention times to find which lipid species from the database were present in the current data. One of these matched lipid species was used to define an "anchor point" for the rest of the lipid species in the lipid class. The model for the retention time was defined by three

main parameters: rt_delta1, rt_delta2, the anchor point, as well as a transformation retention time. First, the retention times were transformed according to this function. Different transformations were used for different lipid classes, specifically, base 2 log transformation for all lipids except triglycerides, and base 2 exponential transformation for triglycerides, because the corresponding elution region had a steeper slope. Parameter rt_delta1 corresponded to the shift in transformed retention time when one double bond was added to the lipid species, and rt_delta2 corresponded to the shift in transformed retention time when one carbon was added to the lipid species. Starting from the anchor point, this model defined the predicted retention time for any lipid species of the lipid class. Defining additional parameter rt_error, we used this model to annotate the lipid features by keeping the lipid features with retention times within +- rt_error boundary of the predicted retention times, and discarding the rest.

Next, the model was trained on the data to find optimal parameters rt_delta1 and rt_delta2. To do this, we defined the score function as the number of annotated lipid features. We performed a simple grid search over an array of rt_delta1 and rt_delta2 to find the optimal parameters. Using these parameters, we annotated the lipid features as described above. This procedure was data-based, and the parameters were not necessarily ideal. For this reason, the resulting putative annotation based on m/z values and retention times were additionally manually curated.

For PC-O and PC-P lipid classes, as well as PE-O and PE-P classes, a modification of the procedure was used. Because these lipid classes have identical m/z values and similar retention times, the above described algorithm could not separate them. In the modified procedure, we similarly started with an anchor point for the -O lipid class and an anchor point for the -P lipid class, but used the optimal parameters rt_delta1 and rt_delta2 calculated for the corresponding PC or PE class.
