# Supplementary material for: Long-Term Fluoxetine Administration Causes Substantial Lipidome Alteration of the Juvenile Macaque Brain
Source: Int J Mol Sci. 2021 Jul 28;22(15):8089. doi: 10.3390/ijms22158089 (PMC8348031; doi:10.3390/ijms22158089)
Supplement: Supplementary file 1 [file ijms-22-08089-s001.zip › Table_legends.pdf]

Long-term fluoxetine administration causes substantial lipidome alteration of the juvenile macaque brain

Anna Tkachev[1,2,§], Elena Stekolshchikova[1,§], Daniil M. Bobrovskiy[3], Nickolay Anikanov[1], Polina Ogurtsova[1], Dong Ik Park[4,#], Anja K.E. Horn[5], Daria Petrova[1], Ekaterina Khrameeva[2,*], Mari S. Golub[6,*], Christoph W. Turck[4,*], Philipp Khaitovich[1,*]

[1] Center for Neurobiology and Brain Restoration, Skolkovo Institute of Science and Technology, Moscow, 121205, Russia
[2] Center of Life Sciences, Skolkovo Institute of Science and Technology, Moscow, 121205, Russia
[3] Faculty of Bioengineering and Bioinformatics, Moscow State University, Moscow 119234, Russia
[4] Max Planck Institute of Psychiatry, Munich, Germany
[5] Ludwig Maximilians University, Department I, Institute of Anatomy and Cell Biology, Munich, Germany
[6] California National Primate Research Center, University of California, Davis, CA, USA

[§] Contributed equally

[*] Correspondence should be addressed to: e.khrameeva@skoltech.ru, msgolub@ucdavis.edu, turck@psych.mpg.de, and khaitovich@eva.mpg.de

[#] Present address:

Aarhus University, Department of Biomedicine, Ole Worms Allé 3, 8000 Aarhus C, Denmark

Supplementary Table S1.
Sample information

Supplementary Table S2.
Gene expression table

Supplementary Table S3.
Table of polar metabolite abundances

Supplementary Table S4.
Table of lipid abundances

Supplementary Table S5.
Polar metabolite information, including annotation and p-values for t-test of fluoxetine vs control groups.

Supplementary Table S6.
Lipid information, including annotation and p-values for t-test of fluoxetine vs control groups

Supplementary Table S7.
Gene information, including p-values for t-test of fluoxetine vs control groups

Supplementary Table S8.
Results for lipid class analysis (Gene Set Enrichment Analysis method)

Supplementary Table S9.
Table of lipid enzymes corresponding to the lipid classes

Supplementary Table S10.
Gene Set Enrichment Analysis (GSEA) results for gene expression

Supplementary Table S11.
Table of links from KEGG compounds to LMSD identifiers.
